# LAG3 genotype of the donor and clinical outcome after allogeneic transplantation from HLA-identical sibling donors

**DOI:** 10.3389/fimmu.2023.1066393

**Published:** 2023-01-20

**Authors:** David Cruz, Rocío Rodríguez-Romanos, Marta González-Bartulos, Irene García-Cadenas, Rafael de la Cámara, Inmaculada Heras, Ismael Buño, Nazly Santos, Natàlia Lloveras, Pilar Velarde, Esperanza Tuset, Carmen Martínez, Marcos González, Guillermo F. Sanz, Christelle Ferrá, Antonia Sampol, Rosa Coll, Jose A. Pérez-Simón, Javier López-Jiménez, Manuel Jurado, David Gallardo

**Affiliations:** ^1^ Hematology Department, Institut Català d’Oncologia - Hospital Dr. Josep Trueta, Institut d’Investigació Biomèdica de Girona (IDIBGI), Josep Carreras Research Institute, Girona, Spain; ^2^ Hematology Department, Hospital de la Santa Creu i Sant Pau, Institut d’Investigació Biomèdica Sant Pau, Universitat Autónoma de Barcelona, Barcelona, Spain; ^3^ Hematology Department, Hospital La Princesa, Madrid, Spain; ^4^ Hematology Department, Hospital General Universitario Morales Meseguer, Murcia, Spain; ^5^ Hematology Department and Genomics Unit, Hospital General Universitario Gregorio Marañón, Gregorio Marañón Health Research Institute (IiSGM), Complutense University, Madrid, Spain; ^6^ Department of Medicine, Universitat de Girona, Girona, Spain; ^7^ Hematology Department, Hospital Clínic de Barcelona, Institut d’Investigacions Biomèdiques August Pi i Sunyer (IDIBAPS), Barcelona, Spain; ^8^ Hematology Department, Hospital Clínico Universitario, Salamanca, Spain; ^9^ Hematology Department, Hospital Universitari i Politècnic La Fe, Valencia, Spain; ^10^ Hematology Department, Institut Català d’Oncologia – Hospital Germans Trias i Pujol, Josep Carreras Research Institute, Badalona, Spain; ^11^ Hematology Department, Hospital Universitari Son Espases, IdISBa, Palma de Mallorca, Spain; ^12^ Hematology Department, Hospital Universitario Virgen del Rocío, Instituto de Biomedicina de Sevilla, Universidad de Sevilla, Sevilla, Spain; ^13^ Hematology Department, Hospital Universitario Ramón y Cajal, Madrid, Spain; ^14^ Hematology Department, Hospital Universitario Virgen de las Nieves, Granada, Spain

**Keywords:** LAG3: lymphocyte activation gene 3, graft-versus-host disease, allogeneic transplant, immune response, checkpoint molecules

## Abstract

**Introduction:**

The association of polymorphisms in molecules involved in the immune response (checkpoint inhibitors) with the clinical outcome after allogeneic transplantation (alloHSCT) has been described. Lymphocyte Activation 3 (LAG3) is a surface protein that plays a regulatory role in immunity as an inhibitory immune checkpoint molecule.

**Methods:**

To determine its role in the alloHSCT setting, we analyzed 797 patients transplanted from HLA-identical sibling donors. The LAG3 rs870849 C>T polymorphism was genotyped in donors.

**Results:**

We detected a higher incidence of severe acute GVHD in patients transplanted from donors with TT genotype (p: 0.047, HR 1.64; 95% CI 1.01 – 2.67). Overall survival (OS) was worse for patients transplanted from donors with the rs870849 CT/TT genotype (0.020; HR, 1.44; 95% CI 1.06 – 1.96), as well as disease-free survival (DFS) (p: 0.002; HR 1.58, 95%CI: 1.18 – 2.14) and transplant-related mortality (TRM) (p< 0.001; HR: 1.88, 95% CI 1.29 – 2.74). When combining the LAG3 rs870849 and the PDCD1 rs36084323 genotypes of the donor, three genetic groups were well defined, allowing a good stratification of the risk of acute GVHD, TRM, OS and DFS.

**Discussion:**

We conclude that the LAG3 genotype of the donor may be considered in donors’ selection. As this selection may be limited in the HLA-identical sibling donor scenario, further studies exploring the impact of LAG3 genotype of the donor in unrelated transplantation are warranted.

## Introduction

Genetic polymorphisms in genes coding for molecules responsible for costimulatory or inhibitory effects on T-lymphocytes have been reported to be associated with autoimmune diseases and susceptibility to tumors (1, 2). It has also been explored the correlation between these polymorphisms and the appearance of GvHD after allogeneic stem cell transplant. In this sense, specific genetic variants in both the cytotoxic T-lymphocyte antigen 4 (*CTLA4*) gene, and the programmed cell death 1 (*PD-1)* gene of the donor have been associated with differences in the clinical outcome after allogeneic hematopoietic stem cell transplantation (alloHSCT) (3–5).

Lymphocyte Activation 3 (LAG-3), also known as CD223, belongs to the immunoglobulin superfamily and contains 4 extracellular immunoglobulin domains and is expressed in T lymphocytes, B lymphocytes, NK cells, and plasmacytoid dendritic cells. The LAG3 gene is located next to CD4 in chromosome 12 and contains 8 exons (6). LAG3 transcription is upregulated following TCR activation and associated cytokine release (IL-2, IL-12, IL-10, IFN gamma) (7–9).

Structurally, LAG-3 is a CD4-homologous protein, although it shares only 20% of the aminoacidic sequence. Following lymphocyte activation, LAG3 interacts with molecules of the major histocompatibility complex of class II (MHC-II) by a few residues of the D1 domain, in the most distal part of the cell membrane of the lymphocyte, generating an inhibitory signal through its intracellular portion and therefore, bringing the lymphocyte to a state of anergy (10–12). In addition to MHC-II, other LAG3 ligands have been discovered: galectine 3 (Gal-3), fibrinogen-like protein 1 (FLG-1), and its overexpression on cancer cells have been identified as a mechanism of tumor immune evasion (13, 14).

LAG3 is stored in the trans-Golgi vesicles (late endosome) of CD8+ lymphocytes together with PD-1, and the action of the two molecules is synergistic by inhibiting lymphocyte activation (15, 16).

Like CTLA-4, LAG3 has two main isoforms. The transmembrane form, of 70 kDa, is formed by the 4 immunoglobulin domains (D1-D4) associated with the intracytoplasmic portion (KIELLE) which will deliver the inhibitory signal. The soluble form of LAG3 (sLAG3) is derived from the proteolytic cleavage of surface LAG3 mediated by disintegrin and metalloproteinase ADAM10 and ADAM17 at the connecting peptide between D4 and the transmembrane domain (17). This soluble form of LAG3 has no known biological function, and the cleavage by itself seems to be a post-translational regulation of membrane LAG3 expression (18).

LAG-3 has emerged as an important regulatory molecule of the immune response, comparable to CTLA-4 or PD-1, consisting in the inhibition of T-cell activation. As it has been described for PD-1, constitutive expression of LAG-3 is associated with exhausted T cells and Treg. The LAG-3 gene has 8 exons, and several single-nucleotide polymorphisms (SNPs) have been described. The C/T transition encoded by the SNP rs870849 encodes a substitution of the amino acid isoleucine for the amino acid threonine in the transmembrane domain of the LAG-3 protein (Thr455Ile). Zhang et al. described an association between the LAG-3 rs870849 TT genotype and an increased risk of multiple sclerosis (19), but these results have not been validated by other groups (20).

To date, there are not data exploring whether LAG-3 may be a molecule involved in the development of GVHD in patients receiving an allogeneic stem cell transplant. This study aims to investigate whether donor’s LAG-3 genotype influences clinical outcome after alloHSCT from HLA-identical sibling donors.

## Patients and methods

### Patients

We retrospectively studied a cohort of 797 patients receiving an allogeneic HSCT from an HLA-identical sibling donor between 2000 and 2014 in Spanish centers. The conditioning regimen was myeloablative in 70.1% of cases and peripheral blood was the most used stem cells source. The clinical characteristics of the studied cohort are summarized in **Table 1**. DNA isolated from peripheral blood from the HSC donors was analyzed for the *LAG3* genotype.

**Table 1:** Clinical characteristics of the patients included in the study according to the LAG3 genotype of the donor (n: 797).

	All cases	CC	CT	TT	p
Median age (range)	42.0 (2 – 65)	45.5 (2 – 65)	43.0 (4 – 65)	38 (4 – 65)	0.447
Sex (Male/Female)	488 (61.2%) 309 (38.8%)	177 (61%) 113 (39%)	229 (61.1%) 146 (38.9%)	82 (62.1%) 50 (37.9%)	0.974
Male recipient - Female donor	192 (24.1%)	60 (20.7%)	88 (23.5%)	44 (33.3%)	0.018
Diagnosis					
Acute lymphoblastic leukemia Acute myeloid leukemia Chronic myeloid leukemia Non-Hodgkin’s lymphoma Myelodysplastic syndrome Severe aplastic anemia Other	134 (16.8%) 279 (35.0%) 106 (13.3%) 151 (18.9%) 83 (10.4%) 34 (4.3%) 10 (1.2%)	43 (14.8%) 100 (34.5%) 42 (14.5%) 57 (19.7%) 31 (10.7%) 10 (3.4%) 7 (2.4%)	60 (16%) 141 (37.6%) 48 (12.8%) 71 (18.9%) 36 (9.6%) 17 (4.5%) 2 (0.5%)	31 (23.5%) 38 (28.8%) 16 (12.1%) 23 (17.4%) 16 (12.1%) 7 (5.3%) 1 (0.8%)	0.110
Advanced disease (beyond CR1)	270 (33.9%)	91 (34.5%)	136 (40.2%)	43 (37.4%)	0.349
Source of stem cells (PB)	599 (75.1%)	225 (77.6%)	273 (73%)	100 (75.8%)	0.391
Myeloablative conditioning regimen	558 (70%)	184 (64.6%)	266 (71.9%)	94 (73.4%)	0.074
Total body irradiation	258 (32.4%)	91 (32%)	127 (34.4%)	40 (31.3%)	0.730
GvHD prophylaxis					0.273
Cyclosporine + Methotrexate Other combinations	557 (69.9%) 240 (30.1%)	199 (68.6%) 91 (31.4%)	258 (68.8%) 117 (31.2%)	100 (75.8%) 32 (24.2%)	

CR1, first complete remission; PB, peripheral blood.

Comparison between genetic groups.

Written informed consent was obtained from patients and donors before DNA storage. DNA samples were obtained from peripheral blood using the QIAamp DNA Blood Mini Kit (Qiagen, GmbH, Hilden, Germany), in accordance with the manufacturer’s instructions, and stored at -80°C until use. Samples from patients included in this study were provided by the IDIBGI Biobank (Biobanc IDIBGI, B.0000872), integrated in the Spanish National Biobanks Network and they were processed following standard operating procedures with the appropriate approval of the Ethics and Scientific Committees. The study was approved by the Ethics Committee of the Dr. Josep Trueta University Hospital (Girona, Catalonia, Spain) and fulfilled the recommendations of the Helsinki declaration.

### LAG3 genotyping

According to the allele frequency and previous association with autoimmune diseases, we decided to explore the rs870849 C>T polymorphism, located in the transmembrane domain.

The genotype of the donors for this polymorphism was determined *via* allelic discrimination plots on the QuantStudioTM 6 and 7 Flex Real-Time PCR System (Applied Biosystem, Carlsbad, CA) by using TaqMan real-time PCR primers and probes obtained as commercially available (TaqMan SNPmGenotypingAssay (rs870849); reference number: 4351379 (Applied Biosystem, Carlsbad, CA). The Assay reagents included both the necessary primers and fluorescently labeled (FAM and VIC) TaqMan MGB probes to amplify and detect each polymorphism. The PCR cycling conditions were as follows: initial denaturation at 95°C for 10 min, followed by 40 cycles of denaturation at 95°C for 15 s and annealing/extension at 60°C for 60 s. The presence of wild-type and variant alleles was defined by comparing the relative end-point fluorescence created by the degradation of each fluorescently labeled TaqMan probe, according to the manufacturer’s instructions.

### Statistical analysis

Allele and genotypes frequencies were formulated by direct counting.

Homogeneity between genotype groups was performed using the Chi-square test for qualitative variables and Student’s T-test or ANOVA for continuous variables.

Univariate analyses were performed to evaluate the association between the rs870849 genotype of the donor and acute GVHD (aGVHD), overall survival (OS), disease-free survival (DFS), Transplant-related mortality (TRM) and relapse.

The Kaplan-Meier method was applied for the analysis of OS and DFS. Curves were compared using the log-rank test. Cumulative incidence estimates were used to explore differences in aGVHD, TRM and relapse. Death without signs of aGVHD was considered as a competitive risk for acute GVHD. The competing risk for TRM was death after relapse and the competing risk for relapse was death in complete remission. Patients with non-malignant diseases were excluded in the relapse analysis. Chronic GVHD incidence was considered as a qualitative variable instead a time-dependent one because the onset of this complication was often missing or not clear, comparing incidence between genetic groups by the chi squared test in univariate analysis and logistic regression model in multivariate analysis.

Multivariate analysis with regression modeling of competing risks (21) was performed to analyze the effects of the LAG-3 genotype of the donor and other factors on aGVHD, TRM and relapse. Multivariate analysis using Cox proportional hazard model was performed for overall survival and DFS. A two-sided p value below 0.05 was considered to be statistically significant.

## Results

### LAG3 genotype distribution

*LAG3* rs870849 genotype could be successfully determined in all the analyzed donors. As previously described in Caucasian population, the C allele was the majoritarian, being detected in 83.5% of cases whereas the T allele was identified in 63.7% of the studied cases. The genotype distribution showed 290 donors (36.4%) homozygous for the C allele, 132 homozygous for the T allele (16.6%) and 375 heterozygous CT (47.1%).

Distribution of relevant clinical variables was comparable between groups based on the *LAG3* rs870849 genotype of the donor except a higher proportion of patients with the combination Male recipient - Female donor in the group with donors homozygous for the T allele (**Table 1**).

### LAG3 genotype of the donor *and clinical outcome after allogeneic hematopoietic transplant from HLA-identical sibling donors*

All patients showed successful engraftment. The cumulative incidence of grades II, III or IV acute GvHD at 180 days was similar in patients receiving grafts from donors that were homozygous for the *LAG3* rs870849 C allele (34.9%), heterozygous CT (32.5%) or homozygous for the T allele (33.8%) (univariate analysis showing p: 0.829 and multivariate analysis p: 0.768).

**Figure 1:**
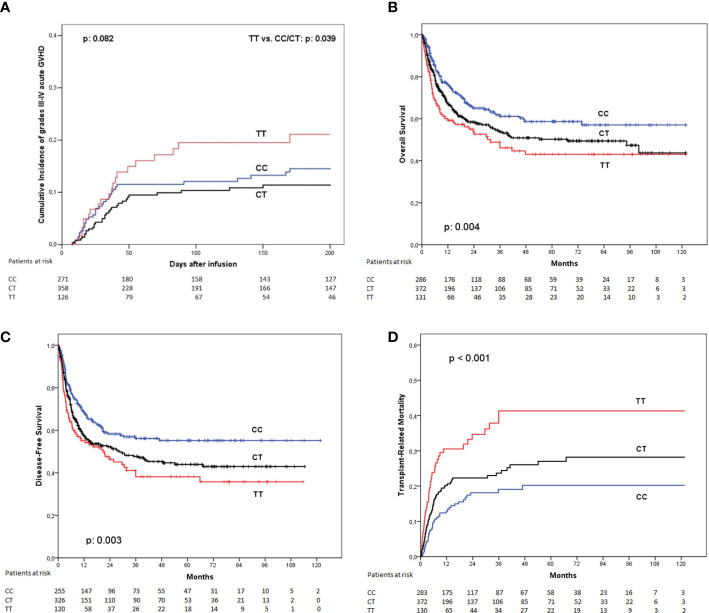
Clinical outcome according to the *LAG3* rs870849 genotype of the donor. **(A)** Incidence of grades III to IV acute graft-versus-host disease, **(B)** Overall survival; **(C)** Disease-free survival and **(D)** Transplant-related mortality.

When considering only severe, grades III or IV acute GVHD at 180 days, the incidence was 13.7% for patients receiving grafts from homozygous rs870849 CC donors, 10.3% for CT genotype, and 18.6% for patients with donors homozygous for the T allele (p: 0.082). Patients transplanted from *LAG3* rs870849 TT donors had a higher incidence of grades III or IV aGVHD when compared with any other genotype (considering together CC and CT genotypes): 18.6% vs 11.8% respectively (**Figure 1A**). Multivariate analysis with regression modeling of competing risks identified the *LAG3* rs870849 TT genotype of the donor as an independent risk factor for developing severe acute GVHD (p: 0.047; hazard ratio [HR]: 1.64, 95% confidence interval [CI] 1.01 – 2.67).

The incidence of chronic GvHD was similar between the *LAG3* rs870849 genetic groups (CC 36.8%; CT 42.7%; TT: 40.2%: p: 0.402).

The presence of a *LAG3* rs870849 T allele in the donor was associated with a significant reduction in overall survival at 5 years. This association was dependent of the number of T alleles of the donor (OS at 5 years: CC: 58.7%, CT: 50.2%, TT: 43.1%; p: 0.004) (**Figure 1B**). Patients transplanted from *LAG3* rs870849 TT donors had worse OS than those transplanted from donors with any other genotype (pooling together CC and CT genotypes) (43.1% vs. 53.9%; p: 0.014). Multivariate analysis using Cox proportional hazard model confirmed that the *LAG3* rs870849 TT genotype was an independent risk factor for worse OS (p: 0.020; HR, 1.44; 95% CI 1.06 – 1.96).

Disease-free survival at five years was also affected by the *LAG3* rs870849 genotype of the donor (DFS at 5 years: CC: 55.2%, CT: 43.9%, TT: 38.2%; p: 0.003) (**Figure 1C**). When comparing DFS between patients transplanted from homozygous TT donors versus any other genotype we detected statistically significant differences in the Cox proportional hazard model (p: 0.002; HR 1.58, 95%CI: 1.18 – 2.14). Evaluation of statistical interaction between the LAG3 TT genotype and the use of female donors for male recipients did not detect significance in multivariate analysis (p: 0.279; HR 1.38 95%CI: 1.06 – 1.81).

This association with OS and DFS was linked with an increased incidence of transplant-related mortality at 5 years in univariate analysis (CC: 20.2%, CT: 27%, TT: 41.3%; p< 0.001) (**Figure 1D**). The TRM incidence of patients receiving transplant from donors with rs870849 TT genotype was higher than those receiving grafts from a donor with any other genotype (CC or CT): 41.3% vs. 24.1% in multivariate analysis with regression modeling of competing risks; (p< 0.001; HR: 1.88, 95% CI 1.29 – 2.74).

**Table 2** shows the detailed univariate and multivariate analysis for severe GVHD incidence, OS, DFS and TRM.

**Table 2:** Univariate and multivariate analysis for acute GVHD incidence, overall survival, disease-free survival and transplant-related mortality.

Grades III to IV acute GVHD	Univariate	Multivariate
Patient’s age	p: 0.554; HR: 1.01 (95% CI 0.99 – 1.03)	p: 0.220; HR: 1.01 (95% CI 0.99 – 1.03)
Diagnosis acute leukemia	p: 0.130; HR: 0.72 (95% CI 0.47 – 1.10)	p: 0.075; HR: 0.68 (95% CI 0.45 – 1.04)
Sex mismatch (male patient/female donor)	p: 0.146; HR: 1.41 (95% CI 0.88 – 2.24)	p: 0.380; HR: 1.24 (95% CI 0.77 – 2.00)
Source of stem cells (peripheral blood)	p: 0.319; HR: 1.30 (95% CI 0.77 – 2.19)	p: 0.180; HR: 1.51 (95% CI 0.82 – 2.79)
Myeloablative conditioning regimen	p: 0.893; HR: 1.03 (95% CI 0.65 – 1.64)	p: 0.220; HR: 1.39 (95% CI 0.82 – 2.37)
T-cell depletion	p: 0.187; HR: 0.55 (95% CI 0.22 – 1.35)	p: 0.150; HR: 0.48 (95% CI 0.18 – 1.31)
Prophylaxis other than cyclosporine + MTX	p: 0.120; HR: 0.71 (95% CI 0.46 – 1.10)	p: 0.076; HR: 0.67 (95% CI 0.44 – 1.04)
Donor with *LAG3* rs870849 TT genotype	p: 0.039; HR: 1.67 (95% CI 1.02 – 2.73)	p: 0.047; HR: 1.64 (95% CI 1.01 – 2.67)
Overall survival		
Patient’s age	p: 0.003; HR: 1.02 (95% CI 1.01 – 1.03)	p< 0.001, HR 1.03 (95% CI: 1.02 – 1.04)
Gender: male	p: 0.600; HR: 1.06 (95% CI 0.85 – 1.33)	p: 0.611, HR 0.97 (95% CI: 0.72 – 1.29)
Diagnosis acute leukemia	p: 0.001; HR: 1.43 (95% CI 1.15 – 1.80)	p: 0.003, HR 1.47 (95% CI: 1.14 – 1.90)
Advanced disease (beyond CR1)	p<0.001; HR: 2.46 (95% CI 1.94 – 3.12)	p< 0.001, HR 2.29 (95% CI: 1.79 – 2.93)
Sex mismatch (male patient/female donor)	p: 0.083; HR: 1.24 (95% CI 0.97 – 1.59)	p: 0.154, HR 1.12 (95% CI: 0.93 – 1.60)
Source of stem cells (peripheral blood)	p: 0.031; HR: 1.34 (95% CI 1.02 – 1.75)	p: 0.671, HR 0.93 (95% CI: 0.68 – 1.25)
Myeloablative conditioning regimen	p: 0.330; HR: 0.89 (95% CI 0.70 – 1.13)	p: 0.047, HR 1.37 (95% CI: 1.00 – 1.87)
T-cell depletion	p: 0.754; HR: 0.94 (95% CI 0.65 – 1.36)	p: 0.881, HR 0.96 (95% CI: 0.62 – 1.48)
Prophylaxis other than cyclosporine + MTX	p: 0.011; HR: 1.36 (95% CI 1.07 – 1.72)	p: 0.177, HR 1.21 (95% CI: 0.92 – 1.58)
Donor with *LAG3* rs870849 TT genotype	p: 0.014; HR: 1.41 (95% CI 1.07 – 1.85)	p: 0.020, HR 1.44 (95% CI: 1.06 – 1.96)
Disease-free survival		
Patient’s age	p: 0.018; HR: 1.01 (95% CI 1.01 – 1.02)	p< 0.001, HR 1.02 (95% CI: 1.01 – 1.03)
Gender: male	p: 0.800; HR: 0.97 (95% CI 0.78 – 1.21)	p: 0.897, HR 0.94 (95% CI: 0.71 – 1.24)
Diagnosis acute leukemia	p: 0.003; HR: 1.40 (95% CI 1.12 – 1.74)	p: 0.003, HR 1.45 (95% CI: 1.13 – 1.87)
Advanced disease (beyond CR1)	p<0.001; HR: 2.14 (95% CI 1.69 – 2.70)	p< 0.001, HR 2.06 (95% CI: 1.62 – 2.63)
Sex mismatch (male patient/female donor)	p: 0.546; HR: 1.08 (95% CI 0.84 – 1.39)	p: 0.740, HR 1.07 (95% CI: 0.78 – 1.47)
Source of stem cells (peripheral blood)	p: 0.577; HR: 1.07 (95% CI 0.84 – 1.37)	p: 0.107, HR 0.79 (95% CI: 0.60 – 1.05)
Myeloablative conditioning regimen	p: 0.820; HR: 1.03 (95% CI 0.81 – 1.31)	p: 0.024, HR 1.44 (95% CI: 1.05 – 1.98)
T-cell depletion	p: 0.885; HR: 1.02 (95% CI 0.72 – 1.45)	p: 0.649, HR 1.16 (95% CI: 0.79 – 1.70)
Prophylaxis other than cyclosporine + MTX	p: 0.181; HR: 1.18 (95% CI 0.92 – 1.51)	p: 0.772, HR 1.04 (95% CI: 0.77 – 1.40)
Donor with *LAG3* rs870849 TT genotype	p: 0.016; HR: 1.38 (95% CI 1.06 – 1.81)	p: 0.002, HR 1.58 (95% CI: 1.18 – 2.14)
Transplant-related mortality		
Patient’s age	p: 0.003; HR: 1.04 (95% CI 1.02 – 1.05)	p<0.001; HR: 1.04 (95% CI 1.03 – 1.06)
Gender: male	p: 0.327; HR: 1.17 (95% CI 0.85 – 1.60)	p: 0.810; HR: 0.95 (95% CI 0.62 – 1.45)
Diagnosis acute leukemia	p: 0.941; HR: 0.99 (95% CI 0.73 – 1.34)	p: 0.880; HR: 1.03 (95% CI 0.72 – 1.46)
Advanced disease (beyond CR1)	p<0.001; HR: 2.20 (95% CI 1.58 – 3.04)	p<0.001; HR: 1.88 (95% CI 1.34 – 2.62)
Sex mismatch (male patient/female donor)	p: 0.004; HR: 1.59 (95% CI 1.15 – 2.19)	p: 0.013; HR: 1.54 (95% CI 1.09 – 2.17)
Source of stem cells (peripheral blood)	p: 0.156; HR: 1.30 (95% CI 0.90 – 1.88)	p: 0.770; HR: 0.94 (95% CI 0.61 – 1.44)
Myeloablative conditioning regimen	p: 0.383; HR: 0.86 (95% CI 0.62 – 1.20)	p<0.001; HR: 1.88 (95% CI 1.28 – 2.77)
T-cell depletion	p: 0.194; HR: 0.68 (95% CI 0.38 – 1.22)	p: 0.250; HR: 0.69 (95% CI 0.36 – 1.30)
Prophylaxis other than cyclosporine + MTX	p: 0.097; HR: 1.31 (95% CI 0.95 – 1.82)	p: 0.470; HR: 0.86 (95% CI 0.58 – 1.28)
Donor with *LAG3* rs870849 TT genotype	p<0.001; HR: 1.95 (95% CI 1.38 – 2.75)	p<0.001; HR: 1.88 (95% CI 1.29 – 2.74)

Concerning the relapse incidence at 5 years, we did not find statistically significant differences related to the *LAG3* rs870849 genotype of the donor (CC: 26.3%, CT: 23.9%, TT: 33.5%; p: 0.118). Multivariate analysis with regression modeling of competing risks also failed to detect an association between the *LAG3* rs870849 genotype and relapse incidence (p: 0.770; HR 1.07, 95%CI: 0.60 – 1.45).

We performed a specific subgroup analysis in patients with acute myeloblastic leukemia (AML), and we detected that the *LAG3* rs870849 CT/TT genotype of the donor was not associated with relapse (CC: 28% vs. CT/TT: 50.5%; p: 0.310; HR 1.40, 95%CI: 0.73 – 2.69).

Also, we explored whether *LAG3* rs870849 genotype of the donor was associated with relapse in patients with or without acute GVHD but did not find this association neither in patients with aGVHD (p: 0.350) nor in those not presenting this complication (p: 0.634).

### Interaction between LAG3 and *PDCD1 genotypes*

LAG3 is stored together with PDCD1, and its liberation seems to have synergistic action by inhibiting T-lymphocyte activation. As the *PDCD1* genotype of the donor has been previously described as having an impact on clinical outcome after HLA-identical sibling donor transplant, we explored the additive effect of the *LAG3* rs870849 C>T and the *PDCD1* rs36084323 A>G genotypes of the donor on the severe acute GVHD incidence.

The genotype at both SNPs was available for 732 donors. The *LAG3* rs870849 TT genotype was associated to the *PDCD1* rs36084323 GG (n: 119; 16.3% of the total combinations) or AG (n: 4; 0.5%) genotypes. The *LAG3* rs870849 CC genotype was associated to the PDCD1 rs36084323 GG genotype in 249 cases (34%), AG in 10 cases (1.4%) and AA in 5 cases (0.7%). The *LAG3* rs870849 heterozygous CT genotype was associated to the *PDCD1* rs36084323 GG genotype in 328 cases (44.8%), AG in 16 cases (2.2%) and AA in 1 case (0.1%).

The presence of the *LAG3* rs870849 TT genotype was associated with an increased risk of severe acute GVHD independently of the *PDCD1* rs36084323 genotype. According to this data and considering the previously reported results considering the clinical outcome in basis to the *PDCD1* genotype of the donor, we defined three genetic groups in 741 patients: group 1) 132 patients transplanted from donors homozygous for the *LAG3* rs870849 T allele, independently of their *PDCD1* rs36084323 genotype; group 2) 577 patients transplanted from donors genotyped as *LAG3* rs870849 CT or CC and *PDCD1* rs36084323 GG [as the G allele in homozygosis was previously suggested to be associated to a higher incidence of acute GVHD (5)]; and group 3) 32 patients transplanted from donors genotyped as *LAG3* rs870849 CT or CC and *PDCD1* rs36084323 AG or AA. The low number of cases in the last genetic group is related to the previously described low frequency of the *PDCD1* rs36084323 A allele in Caucasian population. **Table S1** (**supplementary material**) shows the clinical characteristics of each genetic group and the homogeneity comparison between groups.

**Figure 2:**
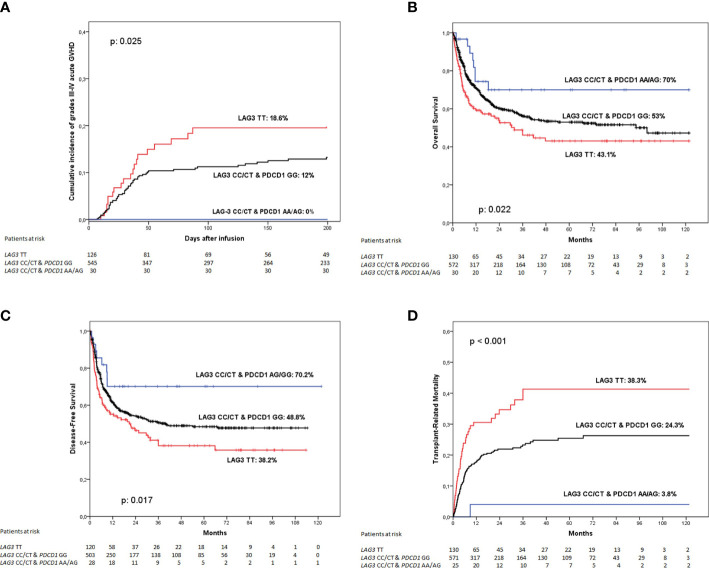
Clinical outcome according to the combined *LAG3* rs870849 and *PDCD1* rs36084323 genotype of the donor. **(A)** Incidence of grades III to IV acute graft-versus-host disease, **(B)** Overall survival; **(C)** Disease-free survival and **(D)** Transplant-related mortality.

When determining the incidence of grades III to IV acute GVHD at 180 days according to these genetic groups we found that patients whose donor was homozygous for the *LAG3* rs870849 T allele had the higher incidence of this complication (18.6%), patients with donors with any other *LAG3* genotype but homozygous for the *PDCD1* rs36084323 G allele had an intermediate incidence (12%) whereas none of the 30 patients whose donors had *LAG3* rs870849 CT or CC and *PDCD1* rs36084323 AG/AA genotypes developed grades III to IV acute GVHD (0%). These differences were globally statistically significant (p: 0.025) (**Figure 2A**). However, comparison between curves showed statistically significant differences only when comparing patients with donors with *LAG3* rs870849 TT genotype and those transplanted from donors genotyped as *LAG3* rs870849 CT or CC and *PDCD1* rs36084323 AG or AA (p: 0.016), whereas no differences were detected when comparing any of these groups with patients transplanted from donors genotyped as *LAG3* rs870849 CT or CC and *PDCD1* rs36084323 GG (p: 0.075 and p: 0.057 respectively). Multivariate analysis confirmed that LAG3 rs870849 TT genotype of the donor was an independent risk factor for a higher incidence of acute GVHD, independently of the PDCD1 genotype (p: 0.031; HR: 1.76, 95%CI: 1.05 – 2.94).

Regarding overall survival, patients receiving grafts from donors with *LAG3* rs870849 TT genotype had poorer outcome than those with other genotypes at this SNP and with *PDCD1* rs36084323 GG genotype, whereas the minority group with *LAG3* rs870849 CT or CC and at least an A allele at *PDCD1* rs36084323 had an excellent OS (43.1%, 53% and 70% respectively; p: 0.022) (**Figure 2B**). When comparing genetic groups face-to-face we detected that patient with donors homozygous for the *LAG3* rs870849 T allele had a statistically significant worse OS than those of group 2 (p: 0.030) or group 3 (p: 0.025), whereas direct comparison between groups 2 and 3 did not show differences (p: 0.132). Multivariate analysis including the PDCD1 genotype identified the donor *LAG3* rs870849 TT genotype as a risk factor for worse OS (p: 0.042; HR: 2.17, 95% CI: 1.03 – 4.58).

As expected, DFS followed a similar pattern (38.2%, 48.8% and 70.2% respectively; p: 0.017) (**Figure 2C**). Again, multivariate analysis considering the PDCD1 genotype detected the *LAG3* rs870849 TT genotype of the donor as an independent risk factor for worse DFS (p: 0.026; HR 2.32, 95%CI: 1.10 – 4.88).

When exploring TRM according to these genetic groups, we found that patients transplanted from donors homozygous for the *LAG3* rs870849 T allele had a 5-years TRM incidence of 38.3%, much higher than those with donors genotyped as *LAG3* rs870849 CT or CC and *PDCD1* rs36084323 GG (24.3%) or *LAG3* rs870849 CT or CC and *PDCD1* rs36084323 AG or AA (3.8%). These differences were statistically significant (p<0.001) (**Figure 2D**). When comparing between curves, we found statistically significant differences between group 1 and 2 (p: 0.001), group 1 and 3 (p: 0.001) and groups 2 and 3 (p: 0.021). Multivariate analysis considering both genetic polymorphisms detected the *LAG3* rs870849 TT genotype as an independent risk factor for increased TRM (p: 0.005; HR: 1.81, 95%CI: 1.20 – 2.73), whereas the PDCD1 rs36084323 of the donor showed no statistical association (p: 0.087; HR: 3.41, 95%CI: 0.84 – 13.88).

## Discussion

Many studies have explored the correlation between genetic variations in genes involved in the inhibition of the T-cell immune response and the development of autoimmune diseases. Moreover, genetic polymorphisms in CTLA-4 and PD-1 genes have been also associated with the incidence of graft-versus-host disease after alloHSCT (3–5).

In the present study, we have examined the relationship between the LAG-3 rs870849 genotype of the donor and the clinical outcome after alloHSCT from HLA-identical sibling donor. Our findings show that patients transplanted from donors homozygous for the T allele at the LAG-3 rs870849 SNP have an increased risk of severe acute GVHD, increased TRM and worse survival. These results agree with the previously described association of the T allele with multiple sclerosis, suggesting that the presence of the T allele impairs the inhibitory potential of the LAG-3 molecule in the allogeneic transplant setting, leading to an increased T-cell response.

It has been speculated that the rs870849 Isoleucine/Threonine (Ile/Thr) substitution would alter the conformational and functional properties of the protein, reducing the binding affinity of LAG3 and leading to an enhanced expansion of T-cells (19). However, this hypothesis seems highly unlikely because this change affects the alpha-helical transmembrane domain.

The relevance of this polymorphism could be explained by looking at the effects driven by the same amino acid polymorphism that has been described within the B cell inhibitory receptor FcgammaRIIB (FCGR2B) gene (rs1050501: FCGR2B c.695T>C) leading to the same amino acid change (Ile/Thr) in the transmembrane helix (22). *In vitro*, the FcgammaRIIB 232Thr showed to be significantly less potent than wild type 232Ile in inhibiting B cell receptor signaling. Moreover, FcgammaRIIB 232Thr was shown to be less effectively distributed to detergent-insoluble lipid rafts, probably due to the substitution of a large hydrophobic amino acid (isoleucine) by a polar one (threonine) (23). If this model may be extrapolated to the LAG3 gene is unknown but is seems plausible to expect that the biological consequences of the rs870849 Ile/Thr change at the transmembrane helix of LAG3 could be similar to those observed in the presence of the FCGR2B rs1050501 Ile/Thr. Although it is well known the critical role of ITIM for the inhibitory function of FcγRIIB, it has been described that the transmembrane domain is also essential for inhibition by impeding the conformational changes of BCR, co-localizing with lipid raft to activate the inhibitory signaling, blocking the synaptic co-localization of BCR and CD19 microclusters and conferring fast lateral mobility (24).

However, if this hypothesis is true, the rs870849 T allele (coding for Isoleucine) should be associated with a higher inhibitory activity of LAG3 and therefore with a lower incidence of acute GVHD in the allogeneic model, which is just opposite to our findings. This inconsistence is difficult to explain. One possible explanation would be a different kinetics of proteolytic cleavage of surface LAG3 mediated by disintegrin and metalloproteinase ADAM10 and ADAM17. *In vitro* studies to correlate the rs870849 genotype with protein expression are currently in development.

It has been reported that LAG-3 and PD-1 molecules have a synergistic effect on T-cell inhibition (16). We explored the clinical outcome after alloHSCT according to the combined LAG-3 rs870849 and PD1.1 rs36084323 genotype of the donor, and we detected that the LAG-3 rs870849 TT genotype is associated with the highest severe acute GVHD incidence and TRM, leading to a worse OS and DFS, independently of the PD-1 rs36084323 genotype. These results suggest that the inhibitory effect of LAG-3 may be stronger than the negative signal driven by PD-1.

This observation may be relevant, as cancer immunotherapy based on immune checkpoint inhibitor blockades is currently one of the main therapeutical approaches for several neoplasms. It is well known that PD-1/LAG-3 expression is a marker of exhausted T-cells infiltrating tumors (25), and high expression of these molecules in T-lymphocytes is associated with poor survival (26). Moreover, it has been suggested that tumors with an immune evasion predominantly mediated by LAG-3 would be less sensitive to immunotherapy with PD-1 inhibitors (nivolumab, pembrolizumab). In this sense, the usefulness of LAG-3 antagonists (Relatlimab) is currently being explored in clinical trials combining both the anti-PD-1 and anti-LAG-3 agents, with promising results (27).

This is the first study correlating the *LAG-3* genotype of the donor with the occurrence of acute GvHD, transplant-related mortality and overall survival after allogeneic transplant in a large cohort of Caucasian patients.

In contrast, a recent Japanese study explored the relationship between transplant outcome and polymorphisms in the immune checkpoint genes, including the LAG3 rs870849 SNP in patients receiving an unrelated alloHSCT, and the authors failed to find any correlation (28). There are some aspects that need to be considered to explain this discrepancy: Takahasi et al. explored Japanese patients transplanted from unrelated donors whereas our study is performed in Caucasian patients transplanted from HLA-identical sibling donors, avoiding hidden or even known HLA mismatches and with less genetic differences potentially influencing the development of GVHD. Moreover, we must consider the differences in the genetic background between European and Asiatic population: the described genetic distribution of the LAG-3 rs870849 T allele is 38.7% in European whereas this allele represents only 17% in East Asia and 14.9% of donors in the Takahasi study, making difficult to detect correlations with the clinical outcome. In this study the number of TT homozygous donors was too small to test the association of LAG3 genotypes with the risk of aGVHD, overall and non-relapse mortality in the recessive model [TT vs (CC and CT)]. However, they did not show any association of donor LAG3 genotypes with the explored endpoints with the allelic (additive) model. Although it is possible that the absence of these associations in the Japanese cohort is ancestry-specific, they raise some significant doubts about the potential clinical application of donor LAG3 genotypes for prediction of outcomes after unrelated HCT. In this sense, new studies looking for the association between LAG3 genotype of the donor and clinical outcome after unrelated transplantation for Caucasian patients should clarify these doubts.

These results need to be confirmed by analysis of independent patient cohorts, but our approach suggests that the routine determination of polymorphisms in genes coding for immune checkpoint inhibitors should be considered when evaluating the predicted clinical outcome associated for each available donor. The limitations of the present study are that we did not account for multiple comparisons and the associations have not been validated by testing in an independent cohort.

The current results could not be applied clinically in donor selection for related alloHSCT, due to the limited availability of HLA-identical siblings, but they potentially could be used in donor selection for unrelated HCT, if these results are reproducible in a similar study with unrelated donors for Caucasian patients.

## Data availability statement

The original contributions presented in the study are included in the article/**Supplementary Materials**, further inquiries can be directed to the corresponding author.

## Ethics statement

The study was approved by the Ethics Committee of the Dr. Josep Trueta University Hospital (Girona, Catalonia, Spain). Written informed consent to participate in this study was provided by the participants’ legal guardian/next of kin.

## Author contributions

DC and DG designed the research, analyzed data and wrote the article; DC, RR-R, MG-B, NS, NL, PV, ET, and RC performed research, IG-C, RdC, IH, IB, CM, MG, GS, CF, AS, JP, JL-J, and MJ participated in the collection and analysis of data. All authors contributed to the article and approved the submitted version.
