# Supplementary material for: LAG3 genotype of the donor and clinical outcome after allogeneic transplantation from HLA-identical sibling donors
Source: Front Immunol. 2023 Jan 20;14:1066393. doi: 10.3389/fimmu.2023.1066393 (PMC9897054; doi:10.3389/fimmu.2023.1066393)
Supplement: Supplementary file 1 [file Table_1.docx]

**Table S1. Homogeneity between genetic groups considering donor LAG3 rs870849 and PDCD1 rs36084323 genotypes**

| LAG3 rs870849 | TT | CT or CC | CT or CC | p |
| --- | --- | --- | --- | --- |
| PDCD1 rs36084323 | any | GG | AG or AA |  |
| Total number | 132 | 577 | 32 | - |
| Median age (range) | 38.0 (4 – 65) | 44.0 (2 – 65) | 32 (14 – 61) | 0.003 |
| Sex (Male/Female) | 82 (62.1%) 50 (37.9%) | 347 (60.1%) 230 (39.9%) | 24 (75.0%) 8 (25.0%) | 0.236 |
| Male recipient - Female donor | 44 (33.3%) | 124 (21.5%) | 12 (37.5%) | 0.003 |
| Diagnosis |  |  |  |  |
| Acute lymphoblastic leukemia  Acute myeloid leukemia  Chronic myeloid leukemia  Non-Hodgkin’s lymphoma  Myelodysplastic syndrome  Severe aplastic anemia  Other | 31 (23.5%)  38 (28.8%)  16 (12.1%)  23 (17.4%)  16 (12.1%)  7 (5.3%)  1 (0.8%) | 90 (15.6%)  208 (36.0%)  76 (13.2%)  111 (19.2%)  62 (10.7%)  23 (4.0%)  7 (1.2%) | 6 (18.8%)  11 (34.4%)  5 (15.6%)  7 (21.9%)  0 (0%)  3 (9.4%)  0 (0%) | 0.176 |
| Advanced disease (beyond CR1) | 43 (37.4%) | 193 (37.2%) | 16 (51.6%) | 0.272 |
| Source of stem cells (PB) | 100 (75.8%) | 435 (75.5%) | 24 (75.0%) | 0.996 |
| Myeloablative conditioning regimen | 94 (73.4%) | 388 (68.4%) | 25 (78.1%) | 0.308 |
| Total body irradiation | 40 (31.3%) | 180 (31.9%) | 17 (53.1%) | 0.041 |
| GvHD prophylaxis |  |  |  | 0.252 |
| Cyclosporine + Methotrexate  Other combinations | 100 (75.8%)  32 (24.2%) | 397 (68.8%)  180 (31.2%) | 21 (65.6%)  11 (34.4%) |  |

CR1: first complete remission; PB: peripheral blood.
